# Standard blood laboratory values as a clinical support tool to distinguish between SARS-CoV-2 positive and negative patients

**DOI:** 10.1038/s41598-021-88844-x

**Published:** 2021-04-30

**Authors:** Rainer Thell, Jascha Zimmermann, Marton Szell, Sabine Tomez, Philip Eisenburger, Moritz Haugk, Anna Kreil, Alexander Spiel, Amelie Blaschke, Anna Klicpera, Oskar Janata, Walter Krugluger, Christian Sebesta, Harald Herkner, Brenda Laky

**Affiliations:** 1 Wiener Gesundheitsverbund, Vienna, Austria; 2 Department of Internal Medicine 2, Emergency Department, Klinik Donaustadt, 122 Langobardenstrasse, 1210 Vienna, Austria; 3 Medical University, Vienna, Austria; 4 Medical School, Sigmund Freud University, Vienna, Austria; 5 Austrian Research Group for Regenerative and Orthopedic Medicine (AURROM), Hartmanngasse 15/10, 1050 Vienna, Austria; 6 Center of Clinical Research, University Clinic of Dentistry, Medical University of Vienna, Vienna, Austria

**Keywords:** Diagnostic markers, Predictive markers

## Abstract

Standard blood laboratory parameters may have diagnostic potential, if polymerase-chain-reaction (PCR) tests are not available on time. We evaluated standard blood laboratory parameters of 655 COVID-19 patients suspected to be infected with SARS-CoV-2, who underwent PCR testing in one of five hospitals in Vienna, Austria. We compared laboratory parameters, clinical characteristics, and outcomes between positive and negative PCR-tested patients and evaluated the ability of those parameters to distinguish between groups. Of the 590 patients (20–100 years, 276 females and 314 males), 208 were PCR-positive. Positive compared to negative PCR-tested patients had significantly lower levels of leukocytes, neutrophils, basophils, eosinophils, lymphocytes, neutrophil-to-lymphocyte ratio, monocytes, and thrombocytes; while significantly higher levels were detected with erythrocytes, hemoglobin, hematocrit, C-reactive-protein, ferritin, activated-partial-thromboplastin-time, alanine-aminotransferase, aspartate-aminotransferase, lipase, creatine-kinase, and lactate-dehydrogenase. From all blood parameters, eosinophils, ferritin, leukocytes, and erythrocytes showed the highest ability to distinguish between COVID-19 positive and negative patients (area-under-curve, AUC: 72.3–79.4%). The AUC of our model was 0.915 (95% confidence intervals, 0.876–0.955). Leukopenia, eosinopenia, elevated erythrocytes, and hemoglobin were among the strongest markers regarding accuracy, sensitivity, specificity, positive and negative predictive value, positive and negative likelihood ratio, and post-test probabilities. Our findings suggest that especially leukopenia, eosinopenia, and elevated hemoglobin are helpful to distinguish between COVID-19 positive and negative tested patients.

## Introduction

In December 2019, an uncommonly high incidence of pneumonia occurred in Wuhan province, China, caused by a previously unknown pathogen and showing an unusually high mortality rate, which showed to be a novel corona virus, SARS-CoV-2 (Severe Acute Respiratory Syndrome—Corona Virus 2)^1^. It causes coronavirus disease (COVID-19) which has reached pandemic levels resulting in significant morbidity and mortality affecting all inhabited areas of the world with large numbers of patients. Hence, the World Health Organisation (WHO) declared a worldwide pandemic in March 2020.

Diagnostic steps for this disease currently are epidemiological contact history, clinical impression, chest radiography, standard blood laboratory, and antigen detection by means of real-time fluorescence PCR. PCR is the gold standard test for detection of SARS-CoV-2 infection^2^.

It soon became apparent that the available test capacities for PCR testing were far from sufficient, and a feverish search for alternative and simpler detection methods began. To date, point-of-care PCR testing is still not available everywhere. Testing that takes a long time can make up for significant additional efforts of organisation of patient cohorts in hospitals.

The clinical appearance of the disease is broadly reported^3^. Signs and symptoms may include fever and cough most commonly, dyspnoea, rarely diarrhoea, anosmia, or ageusia and others, at the beginning or during the course of the disease^4–6^. Various publications indicated that COVID-19 positive patients showed typical laboratory patterns^7–9^, although many of the earlier studies report on relatively small numbers of COVID-19 positive patients.

Zhang et al. included 95 cases with COVID-19-positive pneumonia^7^. They found significantly higher numbers of D-Dimer, C-reactive protein (CRP), and procalcitonin in patients to be admitted to intensive care levels. In a retrospective report of 138 admitted patients, lymphopenia occurred in 70%, a prolonged prothrombin time in 58%, and an elevated lactate dehydrogenase (LDH) resulted in 40% of patients^10^. In an Iranian cohort of 70 COVID-19 positive patients, Mardani et al. found significantly higher neutrophil count, CRP, LDH, aspartate aminotransferase (AST), alanine aminotransferase (ALT), and urea levels, as well as lowered white blood count and albumin levels^11^. A further retrospective trial of 99 patients showed decreased lymphocyte counts in 35%, decreased albumin in 98%, increased LDH in 75%, an increased interleukin-6 in 52%, and raised CRP in 86% of patients were reported^3^. Li et al. performed a trial with 989 patients, detecting a combination of eosinopenia and elevated CRP yielding a sensitivity of 67% and specificity of 78% for COVID-19^12^. In an additional cohort of 458 patients from Guan et al., leukopenia, lymphopenia, eosinopenia, and an increased CRP were detected^4^. In two trials, an increased NLR was described as an independent risk factor of mortality for COVID-19 patients^13,14^.

Standard blood laboratory data are potentially of both, diagnostic and prognostic value^15^. On one hand, they can contribute to judge the pre-test probability of a COVID-19 diagnosis and thus, support the effective and efficacious organisational management of a patient in an emergency department. On the other hand, it is of potential value, if standard blood laboratory blood results can help to distinguish a potentially life-threatening course of a disease from a less critical status.

Therefore, the overall aim of this study was to evaluate standard blood laboratory parameters and clinical characteristics in a large number of COVID-19 suspicious patients, who underwent PCR testing. The specific aim was to determine whether standard blood laboratory parameters are able to identify positive COVID-19 patients from a large COVID-19 suspected cohort.

## Methods

### Data source

Data were obtained from an electronical data base of the Vienna Health Care Association (Wiener Gesundheitsverbund), which stores all medical records of patients treated in its hospitals in Vienna, Austria. Data extraction was exclusively performed by authorised employees of the emergency department at the Klinik Donaustadt, Vienna. The protocol for this retrospective study, including a waiver of the informed consent requirement, was approved both by the Ethics Commission of the City of Vienna (EK 20–122-VK) and the Ethics Commission of Sigmund Freud University Vienna (161/2020). All procedures in this study were performed in accordance with the ethical standards of the institutional and national research committee and with the 1964 Declaration of Helsinki and its later amendments or comparable ethical standards.

### Data collection

Data were collected from female and male adult patients with suspected COVID-19 who underwent reverse transcriptase PCR testing via a nasopharyngeal swab performed at one of the five hospitals (Klinik Donaustadt, Klinik Floridsdorf, Klinik Hietzing, Klinik Landstrasse, and Klinik Ottakring) between February 27, 2020 and April 27, 2020. Based on PCR results, patients were divided into a COVID-19 positive or negative group. Patients without reported standard blood laboratory reports, medical history, or outcome documentation at day 28 after consultation were not included.

As standard blood laboratory testing was performed according to the clinical care needs of the patients, not all parameters were available for all patients. Routine blood tests generally included full blood count, blood chemistry, electrolytes, liver function parameters, renal and myocardial function parameters as well as coagulation markers and markers of inflammation.

Patients’ gender (female/male), age at time of PCR testing (years), coexisting diseases and conditions (e.g. chronic diseases of the lung, liver, kidney; coronary artery disease, diabetes, and arterial hypertension); the clinical 28-day outcome including hospital admission and discharge as well as requirement of intensive care and ventilation, and death details were extracted from medical records.

### Statistical analysis

Descriptive statistics was used to describe the characteristics of patients. Data distribution was determined by visual inspection of the histograms and the Kolmogorov Smirnov tests. Normally distributed data were calculated as mean value with standard deviation (SD), otherwise as median and interquartile range (IQR). Continuous variables were compared between COVID-19 positive and negative patients with independent t-tests (parametric) or Mann–Whitney U-tests (non-parametric). Only those continuous parameters with significant differences were further evaluated. Area under the ROC curves (AUC) were determined in order to assess the ability of continuous blood parameters to distinguish between COVID-19 positive and negative patients.

Blood parameters were categorised according to normal reference ranges used in hospitals. However, since CRP ranges higher than normal (cut off: 0.5 mg/dL) were detected in almost all patients (COVID-19 positive: 99.5% and negative: 98.2%) and thus, useless to discriminate between COVID-19 positive and negative patients, coordinates of the receiver operating characteristic (ROC) and the Youden index were used to determine a, sensitivity and specificity balanced, cut off value for CRP in our cohort. A cut-off for neutrophils-to-lymphocytes ratio (NLR) was also determined using coordinates of the ROC and the Youden index (sensitivity and specificity balanced).

Correlations between the continuous parameters were performed using Kendall’s Tau. Since neutrophils significantly correlated with leucocytes (0.859; *p* < 0.001) and to reduce the number of predictive variables, we used the neutrophils-to-lymphocytes ratio (NLR) instead of neutrophils and lymphocytes. Hemoglobin was used instead of erythrocytes (0.761; *p* < 0.001) and hematocrit (0.874; *p* < 0.001) due to significant correlations. Procalcitonin was also not included due to the small sample size. For further analysis between COVID-19 positive and negative patients, only significant different and clinical meaningful parameters were considered.

Univariate and multivariate binomial logistic regression analyses were used to construct prediction models using PCR results (COVID-19 positive/negative) as the dependent variable and significant patients’ characteristics and blood parameters as predictors (independent variables). Linearity of the continuous variables regarding the logit of the dependent variable was assessed using Box-Tidwell procedure with Bonferroni correction. None of the continuous variables violated the linearity assumption. None of the parameters showed multicollinearity.

Chi-square or Fisher's exact tests were applied to describe the relationship between proportions of categorical variables. Only those categorical parameters with significant differences were further evaluated. Accuracy, sensitivity, specificity, positive predictive value (PPV), negative predictive value (NPV), positive likelihood ratio (LR +), and negative likelihood ratio (LR −) were calculated for each parameter. Bayes’ theorem was used to determine blood parameters’ post-test probabilities, which were calculated using pre-test probabilities and likelihood ratios. Statistical significance was set at a *p* value of < 0.05 (two-sided). All data were analysed with SPSS software (IMP Statistics Version 25; SPSS Inc, Chicago, IL).

## Results

### Characteristics of COVID-19 suspected patients

A total of 655 patients from the four hospitals underwent PCR testing between February 27, 2020 and April 27, 2020. 45 patients were not evaluated due to missing data. Another 17 patients who were tested within this time period with complete data sets were excluded since they were hospitalized more than one month before (n = 16) or more than seven days after (n = 1) PCR-testing. Patients (n = 3) with eosinophilia and acute malignant disease were excluded as well.

The median age of the 276 female (46.8%) and 314 male (53.2%) patients was 71 years (range, 20–100 years; 60.7% of the patients were ≥ 65 years of age at time of PCR testing). No comorbidities were recorded in 69 (33.2%) and 84 (22.0%) COVID-19 positive and negative tested patients, respectively. COVID-19 negative tested patients had significantly more comorbidities than COVID-19 positive tested patients (median (IQR): 1 (1–3) vs. 1 (0–2); *p* < 0.001). The most common comorbidity was pre-existing arterial hypertension (58.1%), followed by diabetes (25.4%), coronary heart disease (19.5%), chronic lung disease (16.9%), chronic kidney disease (15.8%), malignant diseases (13.6%), cerebrovascular accidents (7.5%), chronic liver disease (4.2%), and human immunodeficiency virus (HIV, 0.5%).

A comparison between demographic characteristics and comorbidities between COVID-19 positive and negative tested patients showed no significant differences between the groups (Table 1).

**Table 1:** Comparison of demographic characteristics and 28-day clinical outcome between COVID-19 positive and negative tested patients.

Characteristic	COVID-19 positive (N = 208)	COVID-19 negative (N = 382)	*P* value
Male—no./total no. (%)	117 (56.3)	197 (51.6)	0.276^a^
**Age**			
Median (IQR)—year	72 (57–79.75)	70 (54–81)	0.526^a^
**Comorbidities—no./total no. (%)**			
Hypertension	114 (54.8)	229 (59.9)	0.227*
Diabetes	50 (24.0)	100 (26.2)	0.569*
Coronary heart disease	33 (15.9)	82 (21.5)	0.101*
Chronic lung disease	22 (10.6)	78 (20.4)	0.002*
Chronic kidney disease^b^	29 (13.9)	64 (16.8)	0.371*
Malignant tumor^c^	12 (5.8)	68 (17.8)	< 0.001*
Cerebro vascular accident	13 (6.3)	31 (8.1)	0.410*
Chronic liver disease	2 (1.0)	23 (6.0)	0.004*
Human immunodeficiency virus	0	3 (0.8)	0.556^c^
**Clinical 28-day outcome—no. (%)**			
Not hospitalized/outpatient	23 (11.1)	33 (8.6)	< 0.001*
Discharged after ≤ 28 days hospitalization not requiring ICU support or ventilation	97 (46.6)	245 (64.1)	
Discharged after ≤ 28 days hospitalization requiring ICU support and/or ventilation	4 (1.9)	25 (6.5)	
Still in hospital after day 28	31 (14.9)	44 (11.5)	
Died before day 28	53 (25.5)	35 (9.2)	

Abbreviation: *COVID-19* coronavirus disease 2019, *ICU* intensive care unit, *IQR* interquartile range.

^a^Chi-square test, ^b^Mann–Whitney U-test, ^c^Fischer's exact test.

Similar numbers of outpatients tested positive (11.1%) or negative (8.6%; *p* = 0.338). There was also no significant difference between COVID-19 positive compared to negative tested patients regarding the number of patients requiring ICU and/or ventilation (4.0% vs. 9.3%, respectively; *p* = 0.091). Significantly more COVID-19 positive (25.5%) than negative (9.2%) tested patients died before day 28 (*p* < 0.001). Main causes of death in COVID-19-positive patients were, in descending order, pneumonia (67.3%), followed by multi-organ failure (24.5%), acute cardiac failure (7.6%), and acute renal failure (1.9%).

### Comparison of standard blood laboratory parameters between Covid-19 positive and negative patients

COVID-19 positive patients had significantly lower levels of leukocytes, neutrophils, basophils, eosinophils, lymphocytes, NLR, monocytes, and thrombocytes; while significantly higher levels were detected with erythrocytes, hemoglobin, hematocrit, CRP, ferritin, aPTT, ALT, AST, lipase, CK, and LDH compared to COVID-19 negative patients. Similar levels were detected regarding procalcitonin, albumin, glucose, potassium, total bilirubin, GGT, creatinine, and BUN between the groups (Supplement Table 1).

From all evaluated continuous blood parameters, eosinophils (79.4%), ferritin (76.4%), leukocytes (72.3%), and erythrocytes (72.3%) showed the highest AUC regarding their ability to distinguish between COVID-19 positive and negative patients (Table 2).

**Table 2:** Diagnostic performance of each continuous blood laboratory parameters to distinguish between COVID-19 positive from negative tested patients.

Parameters	AUC (95% CI)	*P* value
Leukocytes (10^9^/L)	0.277 (0.235–0.320)	< 0.001
Neutrophils (10^9^/L)	0.299 (0.252–0.346)	< 0.001
Basophils (10^9^/L)	0.300 (0.253–0.346)	< 0.001
Eosinophils (10^9^/L)	0.206 (0.164–0.249)	< 0.001
Lymphocytes (10^9^/L)	0.376 (0.327–0.425)	< 0.001
Neutrophil-to lymphocyte rate	0.443 (0.391–0.496)	0.032
Monocytes (10^9^/L)	0.394 (0.344–0.445)	< 0.001
Thrombocytes (10^9^/L)	0.423 (0.376–0.470)	0.002
Erythrocytes (10^12^/L)	0.723 (0.681–0.765)	< 0.001
Hemoglobin (g/dL)	0.697 (0.653–0.740)	< 0.001
Hematocrit (%)	0.668 (0.623–0.712)	< 0.001
CRP (mg/dL)	0.610 (0.0563–0.657)	< 0.001
Ferritin (mcg/L)	0.764 (0.660–0.868)	< 0.001
aPTT (s)	0.619 (0.564–0.674)	< 0.001
ALT (U/L)	0.592 (0.541–0.642)	0.001
AST (U/L)	0.657 (0.597–0.716)	< 0.001
Lipase (U/L)	0.684 (0.632–0.735)	< 0.001
CK (U/L)	0.561 (0.510–0.611)	0.023
LDH (U/L)	0.659 (0.607–0.711)	< 0.001

Abbreviations: *ALT* alanine aminotransferase, *aPTT* activated partial thromboplastin time, *AST* aspartate aminotransferase, *AUC* area under the curve, *CK* creatine kinase, *CRP* C-reactive protein, *LDH* lactate dehydrogenase, *NLR* neutrophil-to-lymphocyte ratio.

Comorbidities due to their low discrimination ability (Table 3) were not included in multivariate analyses.

**Table 3:** Univariate analyses and area under receiver operating characteristic curve of comorbidities to distinguish between COVID-19 positive and negative tested patients.

Parameters	OR (95% CI)	*P* value	AUC (95% CI)	*P* value
**Comorbidities**				
Chronic lung disease	0.46 (0.28–0.77)	0.003	0.451 (0.403–0.498)	0.048
Malignant tumor	0.28 (0.15–0.54)	< 0.001	0.440 (0.393–0.487)	0.016
Chronic liver disease	0.15 (0.04–0.65)	0.011	0.475 (0.427–0.523)	0.310

Univariate logistic regression analysis with COVID-19 positive tested patients as the dependent variable revealed that the majority of blood parameters including the dichotomized levels were associated with a positive COVID-19 diagnosis (Table 4).

**Table 4:** Uni- and multivariate analyses of standard blood laboratory parameters with COVID-19 positive tested patients as the dependent variable.

Parameters	Univariate OR (95% CI)	*P* value	Multivariate OR (95% CI)	*P* value
**Blood count**				
Leukocytes (10^9^/L)	0.81 (0.77–0.86)	< 0.001	0.69 (0.58–0.83)	< 0.001
Leukocytes (ref: > 10.0)	1.00			
Leukopenia + normal (≤ 10.0)	4.54 (2.90–7.11)	< 0.001	–	–
Neutrophil-to-lymphocyte ratio	1.00 (0.98–1.02)	0.745	–	–
Neutrophil-to-lymphocyte ratio (ref: > 2.33)	1.00			
≤ 2.33	2.97 (1.74–5.07)	< 0.001	1.72 (0.45–6.58)	0.428
Basophils (10^9^/L)	9.55*10^−18^ (6.02*10^−23^ to 1.51*10^−12^)	< 0.001	0.00 (0.00–1334.76)	0.133
Eosinophils (10^9^/L)	0.00005 (0.000003–0.001)	< 0.001	0.00 (0.00–0.97)	0.011
Eosinophils (ref: ≥ 0.1)	1.00			
Eosinopenia (< 0.1)	5.74 (3.63–9.09)	< 0.001	–	–
Monocytes (10^9^/L)	0.26 (0.13–0.51)	< 0.001	0.93 (0.12–7.06)	0.942
Monocytes (ref: > 0.9)	1.00			
Monopenia + normal (≤ 0.9)	2.30(1.22–4.35)	0.010	–	–
Thrombocytes (10^9^/L)	0.998 (0.997–1.00)	0.013	1.00 (1.00–1.01)	0.272
Thrombocytes (> 370)	1.00			
Thrombopenia + normal (≤ 370)	2.00 (1.10–3.66)	0.024	–	–
Hemoglobin (g/dL)	1.39 (1.27–1.52)	< 0.001	1.63 (1.29–2.07)	< 0.001
Hemoglobin (ref: f: < 11.8; m: < 13.5)	1.00			
Normal + high (f: ≥ 11.8; m: ≥ 13.5)	3.40 (2.38–4.86)	< 0.001	–	–
**Inflammation**				
C-reactive protein (U/L)	1.004 (1.002–1.006)	< 0.001	1.01 (1.00–1.02)	0.025
C-reactive protein (ref: < 22 mg/dL)	1.00			
≥ 22 mg/dL	2.68 (1.81–3.96)	< 0.001	–	–
**Coagulation**				
Activated partial thromboplastin time (s)	1.04 (1.01–1.08)	0.005	0.99 (0.92–1.06)	0.683
**Heart function**				
Creatine kinase (U/L)	1.00004 (0.9999–1.0002)	0.675	–	–
Creatine kinase (ref: ≤ 190)	1.00			
High (> 190)	1.65 (1.12–2.42)	0.011	0.84 (0.28–2.59)	0.767
Lactate dehydrogenase (U/L)	1.0005 (0.9996–1.001)	0.274	–	–
Lactate dehydrogenase (ref: ≤ 250)	1.00			
High (> 250)	2.65 (1.77–3.96)	< 0.001	2.36 (0.71–7.83)	0.159
**Liver function**				
Alanine aminotransferase (U/L)	0.999 (0.997–1.002)	0.519	–	–
Alanine aminotransferase (ref: ≤ 45)	1.00			
High (> 45)	1.66 (1.12–2.47)	0.013	1.65 (0.55–4.96)	0.375
Aspartate aminotransferase (U/L)	1.000 (0.998–1.001)	0.653	–	–
Aspartate aminotransferase (ref: ≤ 35)	1.00			
High (> 35)	2.94 (1.85–4.67)	< 0.001	1.39 (0.44–4.38)	0.573
Lipase (U/L)	1.002 (0.999–1.005)	0.137	–	–
Lipase (ref: ≤ 60)	1.00			
High (> 60)	2.43 (1.50–3.92)	< 0.001	1.44 (0.42–4.97)	0.565
**Renal function**				
Creatinine (ref: 0.5**–**1.2 mg/dL)	1.00		–	–
Low (< 0.5)	0.00	0.999		
High (> 1.2)	1.26 (0.87–1.82)	0.214		

Abbreviation: *CI* confidence interval, *OR* odds ratio.

**Figure 1:**
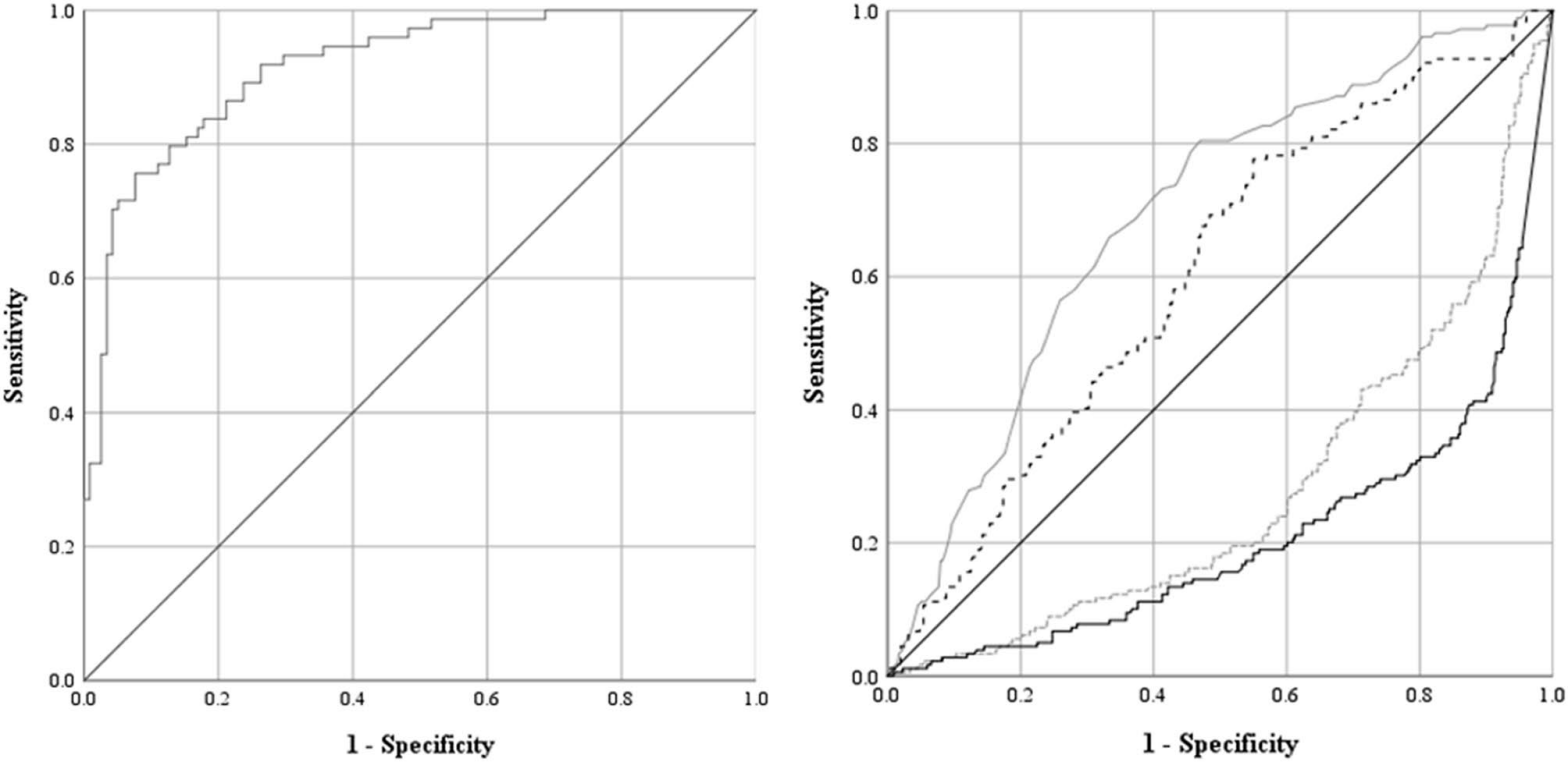
Receiver operating characteristic (ROC) curve of (**A**) the model showing an area under the ROC curve (AUC) of 0.915 (95% confidence intervals (CI), 0.876–0.955) and (**B**) the combined blood parameters leucocytes (doted grey line; AUC = 0.278, 95%CI, 0.232–0.324), eosinophils (straight black line; AUC = 0.208, 95%CI, 0.165–0.250), hemoglobin (straight grey line; AUC = 0.693, 95%CI, 0.646–0.739), and CRP (doted black line; AUC = 0.605, 95%CI, 0.555–0.655).

The multivariate logistic regression model was statistically significant (Chi-square (14) = 119.2; *p* < 0.001) and explained 62.8% (Nagelkerke *R*^2^) of the variance in COVID-19 positive tested patients and correctly classified 83.9% of cases. Sensitivity was 78.4%, specificity was 87.3%, positive predictive value was 79.5%, and negative predictive value was 86.6%. Of the 14 predictors, four were statistically significant including leucocytes, eosinophils, hemoglobin, and CRP (Table 4). Decreasing leucocytes and eosinophils as well as increasing hemoglobin and CRP were associated with an increased likelihood of being COVID-19 positive tested. AUC, as a measure of the overall discriminatory ability of the model, and the model’s best blood parameters combined are presented as ROC curves in Fig. 1.

Accuracy, sensitivity, specificity, PPV, NPV, LR+, LR−, as well as pre-test and post-test probabilities are presented in Table 5. Considering LR+ greater than one (e.g. 10 very strong) and LR− less than one (e.g. 0.1 very strong) are progressively stronger, leukopenia, eosinopenia, as well as elevated erythrocytes and hemoglobin were among the strongest regarding meaningful differences/changes from the pre-test probability.

**Table 5:** Diagnostic performance of single standard categorical blood laboratory parameters to distinguish between COVID-19 positive from negative tested patients.

Parameters	Accuracy	Sensitivity	Specificity	PPV	NPV	LR+	LR−	Pre-test probability	Post-test (pos) probability	Post-test (neg) probability	Change in (pos/neg) probability (%)
Leucocytes (≤ 10 10^9^/L)	0.574	0.448	0.849	0.865	0.415	2.96	0.65	0.684	0.865	0.585	18.1/− 9.9
Neutrophils (< 0.10 10^9^/L)	0.543	0.416	0.829	0.846	0.386	2.44	0.70	0.693	0.846	0.614	15.3/− 7.9
Eosinophils (< 0.10 10^9^/L)	0.620	0.468	0.867	0.852	0.500	3.52	0.61	0.620	0.852	0.500	23.2/− 12.0
Lymphocytes (< 1.5 10^9^/L)	0.506	0.393	0.789	0.824	0.341	1.87	0.77	0.715	0.824	0.659	10.9/− 5.6
NLR (≤ 2.33)	0.324	0.310	0.429	0.802	0.077	0.54	1.61	0.882	0.802	0.923	− 8.0/4.1
Monocytes (≤ 0.9 10^9^/L)	0.416	0.361	0.803	0.929	0.151	1.83	0.80	0.876	0.929	0.849	5.2/− 2.7
Erythrocytes (≥ 4.3 10^12^/L)	0.655	0.509	0.784	0.676	0.643	2.36	0.63	0.470	0.676	0.357	20.6/− 11.3
Hemoglobin (f: ≥ 11.8; m: ≥ 13.5 g/dL)	0.639	0.493	0.777	0.676	0.619	2.21	0.65	0.485	0.676	0.381	19.1/− 10.5
Hematocrit (f: > 38.0%; m: 39.5%)	0.651	0.506	0.746	0.565	0.698	1.99	0.66	0.395	0.565	0.302	17.0/− 9.3
CRP (≥ 22 mg/dL)	0.549	0.423	0.785	0.785	0.423	1.97	0.74	0.650	0.785	0.577	13.5/− 7.3
Procalcitonin (≤ 0.5 ng/mL)	0.485	0.382	0.857	0.907	0.276	2.68	0.72	0.785	0.907	0.724	12.2/− 6.0
aPTT (> 32 s)	0.663	0.469	0.730	0.376	0.799	1.74	0.73	0.257	0.376	0.201	11.8/− 5.6
ALT (> 45 U/L)	0.621	0.414	0.702	0.351	0.754	1.39	0.84	0.280	0.351	0.246	7.0/− 3.5
AST (> 35 U/L)	0.632	0.510	0.739	0.629	0.634	1.95	0.66	0.465	0.629	0.366	16.4/− 9.9
Bilirubin total (≤ 1.0 mg/dL)	0.419	0.355	0.800	0.913	0.173	1.78	0.81	0.856	0.913	0.827	5.8/− 2.9
Lipase (> 60 U/L)	0.658	0.517	0.694	0.298	0.851	1.69	0.70	0.201	0.298	0.149	9.7/− 5.2
CK (> 190 U/L)	0.607	0.430	0.685	0.376	0.732	1.37	0.83	0.306	0.376	0.268	7.0/− 3.8
LDH (> 250 U/L)	0.610	0.457	0.759	0.650	0.588	1.90	0.72	0.494	0.650	0.412	15.5/− 8.3
Creatinine (≥ 0.5 mg/dL)	0.376	0.363	1.000	1.000	0.032	–	0.64	0.979	–	0.968	–/− 1.1

Abbreviations: *ALT* alanine aminotransferase, *aPTT* activated partial thromboplastin time, *AST* aspartate aminotransferase, *AUC* area under the curve, *CK* creatine kinase, *CRP* C-reactive protein, *LDH* lactate dehydrogenase, *NLR* neutrophil-to-lymphocyte ratio.

## Discussion

In this trial, we showed that the likelihood of a SARS-CoV-2 infection can be enforced through standard laboratory blood findings to a high degree. Several studies including meta-analyses recently focused on prediction of the severity of the disease derived from blood results^8,9,16^. Our consideration to find a certain blood pattern to diagnose SARS-CoV-2 infection with standard blood parameters has been less studied.

To our knowledge, only three other trials comparing standard blood parameters between positive and negative cases are published to date^11,12,17^. Similar to those studies, our study showed that leukopenia, eosinopenia, elevated erythrocytes and hemoglobin, and ferritin were detected to be among the best standard laboratory parameters to distinguish between COVID-19 positive from negative tested patients. Accordingly, similar patterns have been detected in positive COVID-19 patients with a severe compared to a mild form of the disease^8,9,16^. The major differences of the three studies, which compared COVID-19 positive and negative patients, opposed to the reviews, which reported only on positive tested patients, were documented regarding leucocytes, neutrophils, and hemoglobin (Table 4).

In conformity with other publications^11,12,17^, leucocytes were lower in COVID-19 positive than negative patients at the time of PCR testing, and so were neutrophils and lymphocytes; while severe compared to mild forms of COVID-19 tend to have higher leucocytes and neutrophils. As opposed to our findings, which showed a weak ability of NLR to discriminate between positive and negative (AUC = 0.443); a raised NLR, which evolved from a raised neutrophil count as well as a lowered lymphocyte count, was already shown previously to be a prognostic value for endotracheal intubation and mortality predictor^13,14^. A cut-off of 4.94 was used in the publication by Tatum et al.^14^; above this value, the risk of being artificially ventilated or to die was increased. Notably, 89% of those patients were African Americans. A lower cut-off (2.33) was established in our study, which might be because only 15% of patients had a neutrophil count higher than 7.7 × 10^9^/L. We are however unaware of any study using NLR as a pure discriminator between positive and negative COVID-19 diagnosis.

However, severity of illness appears to be less important regarding the other parameters, especially regarding eosinophils and CRP (Table 6). Like in other publications^12,17^, our data also showed that eosinopenia was one of the significant predictive biomarkers for COVID-19 with a sensitivity of 47% and a specificity of 87%.

**Table 6:** Blood parameter patterns of COVID-19 positive tested patients of various studies.

	Present study COVID-19 positive	COVID-19 positive	Positive COVID-19 severe
Leucocytes	↓	↓^11,12,17^	↑^8,9,16^
Neutrophils	↓	↓^11,12,17^	↑^8,9,16^
Lymphocytes	↓	↓^11,12,17^	↓^8,9,16^
Eosinophils	↓	↓^12,17^	↓^8,9^
Thrombocytes	↓	↓^12^; not sig. ↓^17^	↓^8,9,16^
Hemoglobin	↑	not sig. ↑^12^	↓^8,16^
CRP	↑	↑^11,12,17^	↑^8,9,16^
ALT	↑	↑^12,17^	↑^8,9^
AST	↑	↑^12,17^	↑^8,9,16^
LDH	↑	↑^12,17^	↑^8,9,16^

Abbreviations: *ALT* alanine aminotransferase, *AST* aspartate aminotransferase, *CRP* C-reactive protein, *LDH* lactate dehydrogenase.

Li et al.^12^ and our study showed an increased hemoglobin in COVID-19 positive patients, which is not in accordance with a lowered hemoglobin in patients with severe COVID-19 disease reported by two meta-analyses^8,16^. In our data, the median hemoglobin was 13.5 g/dL, which did not much differ from Li’s data^12^. In several other trials assessing the severity of disease and blood patterns, hemoglobin was shown to be below normal ranges^8,9,16^. It can only be hypothesized why our cohort presented with a comparably high level of hemoglobin. Possibly, a degree of dehydration played a role at the time of presentation in the emergency department. Indeed, an average temperature of 38.0 ± 0.9 °C on presentation in 99 COVID-19 positive and 37.1 ± 1.4 °C in 103 COVID-19 negative patients, which was a significant difference, was detected in a subgroup analysis of 202 of our patients.

Not surprisingly, CRP was significantly elevated in all studies^11,12,17^. In our patients, we set a new cut-off at 22 mg/dL, since the vast majority of patients had increased CRP values.

Similar blood patterns were also detected regarding ALT, AST, and LDH^8,9,12,16,17^.

Brinati et al. included 279 patients and developed a score for SARS-Cov-2 detection with an accuracy between 82 and 86%, and sensitivity between 92 and 95%^18^. Applying our data including age, gender, leucocytes, neutrophils, lymphocytes, monocytes, eosinophils, basophils, thrombocytes, CRP, AST, ALT, GGT, and LDH to Brinati’s tool, a quite high AUC (AUC = 0.709, 95%CI 0.646–0.771; *p* < 0.001), sensitivity (70.4%), specificity (71.3%), and NPV (79.9%), but less promising PPV (59.8%) could be obtained. However, our model including 14 standard laboratory blood parameters reached better diagnostic performances in all areas (AUC = 0.915, 95%CI 0.876–0.955); sensitivity (78.4%); specificity (87.3%), PPV (79.5%), and NNP (86.6%)), although, the most prominent parameters were leucocytes, eosinophils, hemoglobin, and CRP.

The following limitations of the study should be noted. The retrospective design with missing blood parameters are amongst the major limiting factors. Additionally, with the single time point evaluation, we were not able to retrieve information regarding progression of the disease. Furthermore, cytokines, especially interleukin-6, were not routinely measured, which may be better predictors, especially regarding the so-called ‚COVID-19 cytokine storm ‘, to elucidate COVID-19 positive from negative patients. Another fact to consider is the heterogeneity of underlying diseases, which may also contribute to variations in our findings. On the other hand, such a heterogeneity may reflect reality during a pandemic situation best. Eventually, all test quality crucially depends on the quality of the manual specimen acquisition^19,20^. PCR results tend to be more positive in patients with an increased viral load and with a shorter duration of the disease^21^.

Generally, as laboratory equipment supply develops, more PCR point-of-care diagnostics become available. It is nonetheless doubtful that—neither in the near, nor in the far future—PCR will entirely replace standard laboratory testing. Therefore, the question of a blood laboratory pattern, as specific as possible for COVID-19, remains relevant. Our investigation showed that especially leukopenia, eosinopenia, and elevated hemoglobin are among the best markers to distinguish between COVID-19 positive and negative patients. Therefore, such biomarkers could be useful to facilitate rapid triage of potential COVID-19 patients.

## Supplementary Information

Supplementary Information.

## Data Availability

The datasets generated and/or analysed during the current study are available from the corresponding author on reasonable request.
