# Supplementary material for: Standard blood laboratory values as a clinical support tool to distinguish between SARS-CoV-2 positive and negative patients
Source: Sci Rep. 2021 Apr 30;11:9365. doi: 10.1038/s41598-021-88844-x (PMC8087776; doi:10.1038/s41598-021-88844-x)
Supplement: Supplementary file 1 — Supplementary Information. [file 41598_2021_88844_MOESM1_ESM.pdf]

Standard blood laboratory values as a clinical support tool to distinguish between SARS-CoV-2 positive and negative patients

Rainer Thell[1,2,3], Jascha Zimmermann[4], Marton Szell[1,2], Sabine Tomez[1], Philip Eisenburger[1,4], Moritz Haugk[1], Anna Kreil[1], Alexander Spiel[1], Amelie Blaschke[1,2], Anna Klicpera[1,2], Oskar Janata[1,2], Walter Krugluger[1,2], Christian Sebesta[1,2], M.D., Harald Herkner[3], M.D., and Brenda Laky[3,5,*]

[1]Wiener Gesundheitsverbund, Vienna, Austria; [2]Department of Internal Medicine 2, Emergency Department, Klinik Donaustadt, 122 Langobardenstrasse, 1210 Vienna, Austria; [3]Medical University Vienna, Austria; [4]Sigmund Freud University, Medical School, Vienna, Austria; [5]Austrian Research Group for Regenerative and Orthopedic Medicine (AURROM), Hartmanngasse 15/10, 1050 Vienna, Austria

*Corresponding author: Brenda Laky (ORCID) 0000-0003-1198-4132 (e-mail) brenda.laky@meduniwien.ac.at or brenda.laky@aurrom.org

Supplement Table 1. Comparison of Standard Blood Laboratory Parameters between COVID-19 Positive and Negative tested Patients.

Parameters	N*	COVID-19 TOTAL	N*	COVID-19 Positive	N*	COVID-19 Negative	P value	Pattern
Blood count								
White blood cell count or Leucocytes (10^9/L)								
Median (IQR)	585	7.90 (5.70 to 11.10)	207	6.13 (4.80 to 8.08)	378	9.06 (6.59 to 12.45)	<0.001†	↓
Distribution – no./total no. (%)								
Low (<4.0)	44	7.5	24	11.6	20	5.3		
Normal (4.0-10.0)	356	60.9	155	74.9	201	53.2	<0.001‡	⊥ (not ↑)
High (>10.0)	185	31.6	28	13.5	157	41.5		
Neutrophils (10^9/L)								
Median (IQR)	534	5.79 (4.06 to 8.46)	182	4.45 (3.12 to 6.62)	352	6.66 (4.67 to 9.78)	<0.001†	↓
Distribution – no./total no. (%)								
Low (<1.5)	4	0.7	4	2.2	0	0		
Normal (1.5-7.7)	366	68.5	150	82.4	216	61.4	<0.001‡	⊥ (not ↑)
High (>7.7)	164	30.7	28	15.4	136	38.6		
Basophils (10^9/L)								
Median (IQR)	534	0.02 (0.01 to 0.04)	182	0.02 (0.01 to 0.03)	352	0.03 (0.02 to 0.04)	<0.001†	↓
Distribution – no./total no. (%)								
Normal (0.0-0.2)	532	99.6	182	100	350	99.4	0.550§	⊥ (=)
High (>0.2)	2	0.4	0	0	2	0.6		
Eosinophils (10^9/L)								
Median (IQR)	534	0.06 (0.01 to 0.16)	182	0.01 (0.00 to 0.05)	352	0.10 (0.04 to 0.20)	<0.001†	↓
Distribution – no./total no. (%)								
Low (<0.1)	331	62.0	155	85.2	176	50.0		
Normal (0.1-0.5)	195	36.5	26	14.3	169	48.0	<0.001‡	↓
High (>0.5)	8	1.5	1	0.5	7	2.0		
Lymphocytes (10^9/L)								
Median (IQR)	534	1.10 (0.75 to 1.55)	182	0.96 (0.65 to 1.36)	352	1.21 (0.84 to 1.70)	<0.001†	↓
Distribution – no./total no. (%)								
Low (<1.5)	382	71.5	150	82.4	232	65.9		
Normal (1.5-4.5)	151	28.3	31	17.0	120	34.1	<0.001‡	↓ (less =)
High (>4.5)	1	0.2	1	0.6	0	0		
Neutrophil-to-lymphocyte ratio								
Median (IQR)	534	5.29 (3.16-8.90)	534	5.00 (2.83-8.10)		5.44 (3.30-9.25)	0.032†	↓
Distribution – no./total no. (%)								
≤2.33	63	11.8	36	19.8	27	7.7	<0.001‡	more ↓
>2.33	471	88.2	146	80.2	325	92.3		
Monocytes (10^9/L)								
Median (IQR)	534	0.53 (0.36 to 0.74)	182	0.47 (0.30 to 0.64)	352	0.56 (0.40 to 0.77)	<0.001†	↓
Distribution – no./total no. (%)								
Low (<0.1)	3	0.6	0	0	3	0.9		
Normal (0.1-0.9)	465	87.0	169	92.9	296	84.0	0.013‡	⊥ (less ↓)
High (>0.9)	66	12.4	13	7.1	53	15.1		

Supplement Table 1 (cont.). Comparison of Standard Blood Laboratory Parameters between COVID-19 Positive and Negative tested Patients.

Parameters	N*	COVID-19 TOTAL	N*	COVID-19 Positive	N*	COVID-19 Negative	P value	Pattern
Blood count (cont.)								
Platelet count or Thrombocytes (10^9/L)								
Median (IQR)	584	217.00 (166.25 to 282.75)	207	201.00 (161.00 to 252.00)	377	227.00 (169.50 to 299.00)	0.002†	↓
Distribution – no./total no. (%)								
Low (<150)	104	17.8	35	16.9	69	18.3		
Normal (150-370)	414	70.9	157	75.9	257	68.2	0.052‡	⊥ (=)
High (>370)	66	11.3	15	7.2	51	13.5		
Red blood cell count or Erythrocytes (10^{12}/L)								
Median (IQR)	585	4.20 (3.60 to 4.71)	207	4.60 (4.11 to 5.00)	378	3.90 (3.40 to 4.50)	<0.001†	↑
Distribution – no./total no. (%)								
Low (<4.3)	310	53.0	67	32.4	243	64.3		
Normal (4.3-5.7)	270	46.2	136	65.7	134	35.4	<0.001‡	⊥ (not ↓)
High (>5.7)	5	0.8	4	1.9	1	0.3		
Hemoglobin (g/dL)								
Median (IQR)	585	12.50 (10.70 to 14.10)	207	13.50 (12.30 to 14.70)	378	11.75 (10.28 to 13.33)	<0.001†	↑
Distribution – no./total no. (%)								
Low (f: <11.8; m: <13.5)	301	51.5	67	32.4	234	61.9		
Normal (f: 11.8-15.8; m: 13.5-17.2)	279	47.7	138	66.6	141	37.3	<0.001‡	⊥ (not ↓)
High (f: 15.8; m: >17.2)	5	0.8	2	1.0	3	0.8		
Hematocrit (%)								
Median (IQR)	585	37.20 (32.40 to 41.55)	207	39.70 (36.20 to 42.80)	378	35.70 (31.05 to 40.13)	<0.001†	↑
Distribution – no./total no. (%)								
Low (f: <38.0; m: <39.5)	354	60.5	90	43.5	264	69.9		
Normal (f: 38.0-44.0; m: 39.5-50.5)	216	36.9	110	53.1	106	28.0	<0.001‡	⊥ (not ↓)
High (f: >44.0; m: >50.5)	15	2.6	7	3.4	8	2.1		
Inflammation								
C-reactive protein (mg/dL)								
Median (IQR)	586	43.62 (10.10 to 105.00)	205	61.80 (25.45 to 129.50)	381	33.60 (7.25 to 93.56)	<0.001†	↑
Distribution – no./total no. (%)								
Normal (≤0.5)	8	1.4	1	0.5	7	1.8	0.179‡	= (↑)
High (>0.5)	578	98.6	204	99.5	374	98.2		
Distribution – no./total no. (%)								
< 22 mg/dL	205	35.0	44	21.5	161	42.3	<0.001†	↑ (less ↓)
≥ 22 mg/dL	381	65.0	161	78.5	220	57.7		
Procalcitonin (ng/mL)								
Median (IQR)	130	0.13 (0.05 to 0.48)	43	0.12 (0.05 to 0.28)	87	0.16 (0.05 to 0.64)	0.314†	=
Distribution – no./total no. (%)								
Normal (≤0.5)	102	78.5	39	90.7	63	72.4	0.017‡	⊥ (not ↑)
High (>0.5)	28	21.5	4	9.3	24	27.6		

Supplement Table 1 (cont.). Comparison of Standard Blood Laboratory Parameters between COVID-19 Positive and Negative tested Patients.

Parameters	N*	COVID-19 TOTAL	N*	COVID-19 Positive	N*	COVID-19 Negative	P value	Pattern
Blood chemistry								
Albumin (g/L)								
Median (IQR)	362	30.00 (26.00 to 36.00)	55	30.00 (23.00 to 34.00)	307	31.00 (26.00 to 36.00)	0.207†	=
Distribution – no./total no. (%)								
Low (<35.0)	252	69.6	44	80.0	208	67.8		
Normal (35.0-52.0)	110	30.4	11	20.0	99	32.2	0.080§	= (↓)
High (>52.0)	0	0	0	0	0	0		
Ferritin (mcg/L)								
Median (IQR)	117	169.00 (80.50 to 561.00)	20	579.00 (197.5 to 1704.28)	97	149.00 (57.00 to 370.50)	<0.001†	↑↑
Distribution – no./total no. (%)								
Low (<23.0)	4	3.4	0	0	4	4.1		
Normal (23.0-110.0)	39	33.4	3	15.0	36	37.1	0.080‡	= (↑)
High (>110.0)	74	63.2	17	85.0	57	58.8		
Metabolism								
Glucose (mg/dL)								
Median (IQR)	541	112.00 (96.00 to 143.00)	204	112.00 (99.25 to 141.50)	337	112.00 (95.00 to 144.00)	0.272†	=
Distribution – no./total no. (%)								
Low (<70)	13	2.4	4	2.0	9	2.7		
Normal (70-100)	151	27.9	49	24.0	102	30.3	0.232‡	= (↑)
High (>100)	377	69.7	151	74.0	226	67.0		
Electrolytes								
Potassium (mmol/L)								
Median (IQR)	539	4.00 (3.70 to 4.20)	183	4.00 (3.70 to 4.20)	356	3.90 (3.60 to 4.20)	0.149†	=
Distribution – no./total no. (%)								
Low (<3.5)	65	12.1	17	9.3	48	13.5		
Normal (3.5-5.5)	469	87.0	165	90.2	304	85.4	0.285†	= (⊥)
High (>5.5)	5	0.9	1	0.5	4	1.1		
Sodium (mmol/L)								
Median (IQR)	584	138.00 (135.00 to 140.00)	207	136.00 (133.00 to 139.00)	377	138.00 (136.00 to 140.00)	<0.001†	↓
Distribution – no./total no. (%)								
Low (<135)	131	22.4	72	34.8	59	15.7		
Normal (135-150)	449	76.9	133	64.2	316	83.8	<0.001‡	⊥ (more ↓)
High (>150)	4	0.7	2	1.0	2	0.5		

Supplement Table 1 (cont.). Comparison of Standard Blood Laboratory Parameters between COVID-19 Positive and Negative tested Patients.

Parameters	N*	COVID-19 TOTAL	N*	COVID-19 Positive	N*	COVID-19 Negative	P value	Pattern
Coagulation								
Activated partial thromboplastin time (sec)								
Median (IQR)	439	28.00 (25.00 to 32.50)	141	29.90 (27.00 to 33.05)	298	27.00 (24.00 to 31.00)	<0.001†	↑
Distribution – no./total no. (%)								
Low (<21)	10	2.3	3	2.1	7	2.4		
Normal (21-32)	316	72.0	85	60.3	231	77.5	<0.001‡	⊥ (more ↑)
High (>32)	113	25.7	53	37.6	60	20.1		
Liver function								
Alanine aminotransferase (U/L)								
Median (IQR)	517	27.00 (17.00 to 50.00)	171	32.00 (21.00 to 53.00)	346	25.00 (15.00 to 45.00)	0.001†	↑
Distribution – no./total no. (%)								
Normal (≤45)	372	72.0	111	64.9	261	75.4	0.012‡	⊥ (more ↑)
High (>45)	145	28.0	60	35.1	85	24.6		
Aspartate aminotransferase (U/L)								
Median (IQR)	329	33.00 (23.00 to 62.00)	124	47.00 (29.00 to 70.00)	205	26.00 (20.00 to 52.00)	<0.001†	↑
Distribution – no./total no. (%)								
Normal (≤35)	176	53.5	46	37.1	130	63.4	<0.001‡	↑ (not ↓)
High (>35)	153	46.5	78	62.9	75	36.6		
Total Bilirubin (mg/dL)								
Median (IQR)	520	0.60 (0.40 to 0.80)	173	0.60 (0.40 to 0.80)	347	0.50 (0.40 to 0.90)	0.311†	=
Distribution – no./total no. (%)								
Low (<0.3)	47	9.0	11	6.3	36	10.4		
Normal (0.3-1.0)	398	76.5	147	85.0	251	72.3	0.005‡	⊥ (less ↑)
High (>1.0)	75	14.4	15	8.7	60	17.3		
Gamma glutamyl transpeptidase (U/L)								
Median (IQR)	517	43.00 (25.00 to 94.50)	171	46.00 (27.00 to 88.00)	346	41.00 (23.00 to 98.25)	0.186†	=
Distribution – no./total no. (%)								
Normal (≤60)	331	64.0	109	63.7	222	64.2	0.926‡	=
High (>60)	186	36.0	62	36.3	124	35.8		
Lipase (U/L)								
Median (IQR)	433	29.00 (18.00 to 50.50)	151	39.00 (25.00 to 65.00)	282	23.00 (14.00 to 41.00)	<0.001†	↑
Distribution – no./total no. (%)								
Normal (≤60.0)	346	79.9	106	70.2	240	85.1	<0.001‡	⊥ (more ↑)
High (>60.0)	87	20.1	45	29.8	42	14.9		

Supplement Table 1 (cont.). Comparison of Standard Blood Laboratory Parameters between COVID-19 Positive and Negative tested Patients.

Parameters	N*	COVID-19 TOTAL	N*	COVID-19 Positive	N*	COVID-19 Negative	P value	Pattern
Heart function								
Creatine Kinase (U/L)								
Median (IQR)	517	111.00 (53.50 to 232.00)	181	127.00 (63.00 to 243.50)	336	102.50 (50.00 to 216.00)	0.023†	↑
Distribution – no./total no. (%)								
Normal (≤190)	359	69.4	113	62.4	246	73.2	0.011‡	⊥ (more ↑)
High (>190)	158	30.6	68	37.4	90	26.8		
Lactate dehydrogenase (U/L)								
Median (IQR)	451	248.00 (199.00 to 331.00)	157	285.00 (228.00 to 384.50)	294	225.00 (189.00 to 297.75)	<0.001†	↑
Distribution – no./total no. (%)								
Normal (≤250)	228	50.6	55	35.0	173	58.8	<0.001‡	↑ (less ⊥)
High (>250)	223	49.4	102	65.0	121	41.2		
Renal function								
Creatinine (mg/dL)								
Median (IQR)	585	1.00 (0.80 to 1.30)	208	1.00 (0.80 to 1.34)	377	1.00 (0.80 to 1.30)	0.081†	=
Distribution – no./total no. (%)								
Low (<0.5)	12	2.1	0	0	12	3.2		
Normal (0.5-1.2)	401	68.5	139	66.8	262	69.5	0.016‡	⊥ (less ↓)
High (>1.2)	172	29.4	69	33.2	103	27.3		
Blood urea nitrogen (mg/dL)								
Median (IQR)	498	18.00 (12.00 to 28.00)	178	17.50 (13.00 to 28.00)	320	18.00 (12.00 to 28.00)	0.555†	=
Distribution – no./total no. (%)								
Low (<6)	10	2.0	1	0.6	9	2.8		
Normal (6-25)	341	68.5	123	69.1	218	68.1	0.227‡	= (⊥)
High (>25)	147	29.5	54	30.3	93	29.1		

Abbreviations: f, female; IQR, interquartile range; L, liter; m, male
* Number of parameters available.
† Mann-Whitney U-test
‡ Chi-square test
§ Fisher's exact test
